# Outcomes and Risk Factors for Influenza and Respiratory Syncytial Virus Lower Respiratory Tract Infections and Mortality in Patients With Lymphoma or Multiple Myeloma: A 7-Year Retrospective Cohort Study

**DOI:** 10.1093/ofid/ofaf127

**Published:** 2025-03-04

**Authors:** Tali Shafat, Daniel De-la-Rosa-Martinez, Fareed Khawaja, Ying Jiang, Amy Spallone, Marjorie Vieira Batista, Ella Ariza-Heredia, Diana Vilar-Compte, Sairah Ahmed, Melody Becnel, Roy F Chemaly

**Affiliations:** Department of Infectious Diseases, Infection Control, and Employee Health, The University of Texas MD Anderson Cancer Center, Houston, Texas, USA; European Society of Clinical Microbiology and Infectious Diseases Study Group for Respiratory Viruses, Basel, Switzerland; Department of Infectious Diseases, Instituto Nacional de Cancerología, México City, México; Programa de Estudios Combinados en Medicina, Facultad de Medicina, Universidad Nacional Autónoma de México, México City, México; Department of Infectious Diseases, Infection Control, and Employee Health, The University of Texas MD Anderson Cancer Center, Houston, Texas, USA; European Society of Clinical Microbiology and Infectious Diseases Study Group for Respiratory Viruses, Basel, Switzerland; Department of Infectious Diseases, Infection Control, and Employee Health, The University of Texas MD Anderson Cancer Center, Houston, Texas, USA; Department of Infectious Diseases, Infection Control, and Employee Health, The University of Texas MD Anderson Cancer Center, Houston, Texas, USA; European Society of Clinical Microbiology and Infectious Diseases Study Group for Respiratory Viruses, Basel, Switzerland; European Society of Clinical Microbiology and Infectious Diseases Study Group for Respiratory Viruses, Basel, Switzerland; Department of Infectious Diseases, AC Camargo Cancer Center, São Paulo, Brazil; Department of Infectious Diseases, Infection Control, and Employee Health, The University of Texas MD Anderson Cancer Center, Houston, Texas, USA; European Society of Clinical Microbiology and Infectious Diseases Study Group for Respiratory Viruses, Basel, Switzerland; Department of Infectious Diseases, Instituto Nacional de Cancerología, México City, México; Departments of Lymphoma and Myeloma, The University of Texas MD Anderson Cancer Center, Houston, Texas, USA; Departments of Lymphoma and Myeloma, The University of Texas MD Anderson Cancer Center, Houston, Texas, USA; Department of Infectious Diseases, Infection Control, and Employee Health, The University of Texas MD Anderson Cancer Center, Houston, Texas, USA; European Society of Clinical Microbiology and Infectious Diseases Study Group for Respiratory Viruses, Basel, Switzerland

**Keywords:** hematological malignancies, influenza, lymphoma, multiple myeloma, respiratory syncytial virus

## Abstract

**Background:**

Respiratory viral infection (RVI) is a significant complication in patients with hematologic malignancies. While risk factors of lower respiratory tract infections (LRIs) and mortality have been studied in allogeneic hematopoietic cell transplant recipients, data remain limited for patients with lymphoma and multiple myeloma (MM). We investigated outcomes and risk factors of LRI and mortality secondary to respiratory syncytial virus (RSV) or influenza virus (IFV) infections in these populations.

**Methods:**

We performed a retrospective study in adults with lymphoma or MM with RSV or IFV RVIs between 2016 and 2022. Primary outcomes were LRI and all-cause 30- and 90-day mortality.

**Results:**

We analyzed 440 patients with 490 consecutive viral episodes: 297 (61%) with MM and 193 (39%) with lymphoma, 258 (52%) were IFV-related, and 234 (48%) RSV-related (2 coinfections). At presentation, 62% were diagnosed with upper respiratory tract infection (URI) and 38% with LRI. During follow-up, 57% were hospitalized, 8% required intensive care unit transfer, and 20 (4%) died within 30 days. On multivariable analysis, RSV infection (vs IFV), current/former smoking, steroid exposure, lymphopenia (≤200 cells/mL), and high serum creatinine were associated with LRI. MM (vs lymphoma) diagnosis, current/former smoking, lymphopenia, and nosocomial infection were associated with 30-day mortality, whereas LRI (vs URI), current/former smoking, and lymphopenia were associated with 90-day mortality.

**Conclusions:**

We described a high burden of IFV and RSV infections in patients with lymphoma and MM and found risk factors associated with LRI and mortality. These factors could potentially identify high-risk patients, enabling better and prompt management strategies.

## Teaser

A large retrospective study investigated respiratory syncytial virus or influenza virus infections in patients with lymphoma and multiple myeloma. Risk factors for lower respiratory tract infection and mortality were identified, facilitating better management strategies.

Respiratory viral infections (RVIs) pose a significant challenge for patients with hematologic malignancies (HMs), often leading to substantial morbidity and mortality [1, 2]. Early intervention is critical for patients with HM who have respiratory syncytial virus (RSV) or influenza virus (IFV) infections to prevent progression to lower respiratory tract infection (LRI) and mortality [3, 4]. The disease course and risk factors for RSV and IFV infections have been extensively studied among allogeneic hematopoietic cell transplant (HCT) recipients [5, 6]. However, data on patients with lymphoma and multiple myeloma (MM) are limited to small cohort studies [7–9], and there is no validated scoring index to predict outcomes.

The scarce data on IFV and RSV infections in patients with lymphoma and MM showed LRI progression rates of 27% to 31%; hospitalization rates of 56% to 71%, including 7% to 13% intensive care unit (ICU) admissions; and 30- and 90-day mortality rates of 5% and 8% to 10%, respectively [7, 9, 10]. Within the subpopulation of patients with MM and IFV infections, 1 cohort study reported even worse outcomes, with up to 75% of patients experiencing progression to LRI, 42% admitted to the ICU, and 33% dying during hospitalization [10].

Previously identified risk factors for progression to LRI and mortality in patients with HM and RSV infections, including patients with leukemia in particular, were concurrent lymphopenia and neutropenia and lack of ribavirin treatment [9], hypoalbuminemia, hypoxemia at diagnosis, steroid exposure, elevated creatinine levels, and respiratory coinfections [7]. However, risk factors specific to the development of LRI and mortality due to RVI in lymphoma and MM patients are limited.

In this study, we aimed to identify factors associated with LRI and mortality in a large cohort of patients with lymphoma or MM who had RSV or IFV infections. Identifying such prognostic variables could help identify high-risk patients and guide decision-making to facilitate timely treatment, thereby improving outcomes.

## METHODS

### Study Design

We performed a retrospective cohort study involving consecutive adult patients with lymphoma or MM who had IFV or RSV infections between 1 January 2016 and 31 December 2022, at our institution. Patients with respiratory symptoms were tested for respiratory viruses in either the ambulatory or inpatient setting and at the discretion of their clinical providers. The diagnosis of RVI in symptomatic patients was established using the BioFire Film Array Respiratory Panel 2.1 (BioFire Diagnostics, Salt Lake City, Utah) on nasal washes or swabs. We included multiple viral infection episodes for the same patient if they occurred at least 1 month apart and if the patient experienced clinical recovery between them.

Patient data were collected through electronic medical records. Our primary outcomes of interest were LRI at any time point (from presentation or progression) and all-cause mortality at 30 and 90 days. Secondary outcomes included hospitalization, admission to the ICU, oxygen requirements, mechanical ventilation, and mortality attributed to RVI within 30 days. We also investigated RVI trends over time and seasonality, especially in relation to the coronavirus disease 2019 (COVID-19) pandemic era. This study received approval from the MD Anderson Institutional Review Board (IRB# PA15-0002), and a waiver of consent was granted.

### Definitions

Upper respiratory tract infection (URI) was determined when RSV/IFV was detected in samples obtained from the mucosal surfaces of the upper airways, accompanied by upper respiratory tract symptoms such as nasal congestion, cough, rhinorrhea, sinusitis, and pharyngitis and the absence of clinical or radiologic evidence of LRI [11]. LRI was determined when RSV/IFV was detected in samples from the upper or lower airways, along with new or progressive pulmonary infiltrates suggestive of viral infection and at least 1 lower respiratory tract symptom such as cough, sputum production, fever, hypoxia, shortness of breath, and pleuritic chest pain [12]. Probable LRI was defined as above and in addition to RSV/IFV polymerase chain reaction (PCR) detected only in a nasopharyngeal sample, while laboratory-confirmed LRI required the presence of RSV/IFV PCR in bronchoalveolar lavage fluid [11]. Nosocomial infection was defined as a new-onset infection that occurred >48 hours after admission for IFV or >5 days after admission for RSV.

### Statistical Analysis

The χ^2^ or Fisher exact test was used to compare categorical data, as appropriate. The Student *t* test or Mann-Whitney *U* test was used to compare continuous variables, depending on whether the data followed a normal distribution. Logistic regression analysis was used to identify the independent predictors of the primary outcomes, including LRI and 30- and 90-day all-cause mortality. In detail, first, univariable logistic regression analysis was performed. Next, variables with *P* values ≤.15 from their univariable analyses were selected to construct an initial multivariable logistic regression model, and then the full model was reduced to the final model using the backward elimination procedure, ensuring that all the variables remaining in the final model had *P* values <.05. Survival analysis was conducted using Kaplan-Meier curves to depict 90-day mortality, and the log-rank test was used to compare the curves. All the tests were 2-sided with a significance level of .05. The statistical analyses were performed using IBM SPSS Statistics version 25 (IBM Corporation, Armonk, New York) and SAS version 9.4 (SAS Institute, Cary, North Carolina) software.

## RESULTS

### Study Population

We analyzed 440 patients with 490 consecutive viral episodes; 60.6% had multiple myeloma and 39.4% lymphoma. Table 1 depicts the baseline characteristics of patients with HM and RVI by the site of infection. In brief, more patients with LRI, when compared to URI, were older, former smokers, with active malignancy, infected with RSV, had lymphopenia (<200 cells/mL), had neutropenia (<500 cells/mL), and had higher 30- and 90-day all-cause mortality. At presentation, 187 episodes (38.2%) were classified as LRI. Among the 303 (61.8%) URI episodes, 19 (6.3%) progressed to LRI during 30-day follow-up (Supplementary Table 1). Most of the LRI episodes (82.5%) were categorized as probable.

**Table 1.** Baseline Characteristics and Clinical Outcomes of Patients With Hematologic Malignancy and Respiratory Viral Infection, by Site of Infection

Variable^a^	Total (N = 490)	LRI (n = 206)	URI (n = 284)	*P* Value
Demographics				
Age at RVI diagnosis, y, mean ± SD	61.8 ± 13.8	63.9 ± 13.2	60.2 ± 14.0	**.003**
Sex				
Female	216 (44.1)	90 (43.7)	126 (44.4)	.882
Male	274 (55.9)	116 (56.3)	158 (55.6)	
Race/ethnicity				
Non-Hispanic White	277 (56.5)	116 (56.3)	161 (56.6)	.840
Hispanic	88 (18.0)	39 (18.9)	49 (17.3)	
Black	92 (18.8)	40 (19.4)	52 (18.3)	
Asian	29 (5.9)	10 (4.9)	19 (6.7)	
Other	4 (0.8)	1 (0.5)	3 (1.1)	
Smoking status^b^				
Never	314 (64.2)	119 (58.1)	195 (68.7)	.**048**
Former	161 (32.9)	80 (39.0)	81 (28.5)	
Current	14 (2.9)	6 (2.9)	8 (2.8)	
Influenza vaccination (current season)	130 (26.5)	51 (24.8)	79 (27.8)	.449
HM characteristics				
HM diagnosis				
Hodgkin lymphoma	31 (6.3)	11 (5.3)	20 (7.0)	.163
Non-Hodgkin lymphoma	162 (33.1)	60 (29.1)	102 (35.9)	
Multiple myeloma	297 (60.6)	135 (65.6)	162 (57.1)	
Active malignancy at RVI diagnosis	351 (71.6)	167 (81.1)	184 (64.8)	**<.001**
Active antineoplastic treatment at RVI diagnosis	385 (78.6)	174 (84.5)	211 (74.3)	**.007**
Steroid use within 30 d of RVI diagnosis (mg prednisone equivalent)				
Any	296 (60.4)	151 (73.3)	145 (51.1)	**<**.**001**
30-d cumulative steroid dosage, median (IQR)^c^	533 (240–1066)	533 (266–997)	533 (172–1066)	.597
Peak dose of ≤1 mg/kg/d^d^	86 (29.2)	43 (28.5)	43 (29.9)	.794
Peak dose of >1 mg/kg/d^d^	209 (70.8)	108 (71.5)	101 (70.1)	
Previous chest radiotherapy	96 (19.6)	47 (22.8)	49 (17.3)	.126
History of HCT				
None	266 (54.3)	107 (51.9)	159 (56.0)	.395
Autologous	214 (43.7)	93 (45.2)	121 (42.6)	
Allogeneic	10 (2.0)	6 (2.9)	4 (1.4)	
History of CAR-T therapy	36 (7.3)	11 (5.3)	25 (8.8)	.147
RVI clinical course				
Pathogen				.**006**
RSV	232 (47.3)	112 (54.4)	120 (42.3)	
Influenza	256 (52.3)	92 (44.6)	164 (57.7)	
RSV + influenza	2 (0.4)	2 (1.0)	0 (0.0)	
Respiratory viral coinfections (during ±2 wk)^e^	85 (17.3)	40 (19.4)	45 (15.8)	.303
Year of infection				
2016	45 (9.2)	27 (13.1)	18 (6.3)	.132
2017	98 (20.0)	35 (17.0)	63 (22.3)	
2018	73 (14.9)	34 (16.5)	39 (13.7)	
2019	115 (23.5)	47 (22.8)	68 (23.9)	
2020	53 (10.8)	19 (9.2)	34 (12.0)	
2021	36 (7.3)	17 (8.3)	19 (6.7)	
2022	70 (14.3)	27 (13.1)	43 (15.1)	
Time period of infection				
Pre-COVID-19 era (Jan 2016–Feb 2020)	376 (76.7)	158 (76.7)	218 (76.8)	.987
COVID-19 era (Mar 2020–Dec 2022)	114 (23.3)	48 (23.3)	66 (23.2)	
LRI type^c^				
Probable	…	170 (82.5)	…	
Laboratory confirmed	…	36 (17.5)	…	
RVI symptoms				
Cough	428 (87.3)	184 (89.3)	244 (85.9)	.263
Fever	250 (51.0)	116 (56.3)	134 (47.2)	.**046**
Shortness of breath	162 (33.1)	95 (46.1)	67 (23.6)	**<**.**001**
Rhinorrhea	148 (30.2)	40 (19.4)	108 (38.0)	**<**.**001**
Nasal congestion	151 (30.8)	51 (24.8)	100 (35.2)	.**013**
Fatigue	149 (30.4)	69 (33.5)	80 (28.2)	.206
Sore throat	73 (14.9)	26 (12.6)	47 (16.5)	.228
Chills	81 (16.5)	37 (18.0)	44 (15.5)	.468
Headache	54 (11.0)	20 (9.7)	34 (12.0)	.430
Nausea/vomiting	55 (11.2)	25 (12.1)	30 (10.6)	.586
Myalgia	46 (9.4)	16 (7.8)	30 (10.6)	.295
Diarrhea	38 (7.8)	19 (9.2)	19 (6.7)	.301
Chest pain	33 (6.7)	18 (8.7)	15 (5.3)	.132
Arthralgia	14 (2.9)	4 (1.9)	10 (3.5)	.300
Hypoxia at presentation (≤92%) in room air^f^	49 (10.6)	43 (21.1)^g^	6 (2.3)^h^	**<.001**
Nosocomial infection	20 (4.1)	12 (5.8)	8 (2.8)	.097
Bronchoscopy	42 (8.6)	41 (19.9)	1 (0.4)	**<.001**
Lymphopenia (<200 cells/mL)	60 (12.2)	44 (21.4)	16 (5.6)	**<.001**
Neutropenia (<500 cells/mL)	28 (5.7)	19 (9.2)	9 (3.2)	**.004**
Lymphopenia and neutropenia	17 (3.5)	14 (6.8)	3 (1.1)	**.001**
Elevated creatinine (≥1.2 mg/dL)	146 (29.8)	80 (38.8)	66 (23.2)	**<.001**
Antiviral therapy				
Any therapy	390 (79.6)	184 (89.3)	206 (72.5)	**<**.**001**
Ribavirin	142 (29.0)	92 (44.7)	50 (17.6)	**<**.**001**
Oseltamivir^i^	245 (50.0)	91 (44.2)	154 (54.2)	.**028**
IVIG	67 (13.7)	54 (26.2)	13 (4.6)	**<**.**001**
Antiviral timing from symptom onset				
No treatment	100 (20.4)	22 (10.7)	78 (27.5)	**<**.**001**
Within 48 h	139 (28.4)	56 (27.2)	83 (29.2)	
After 48 h	251 (51.2)	128 (62.1)	123 (43.3)	
RVI outcomes				
Hospital admission				
Any	280 (57.1)	180 (87.4)	100 (35.2)	**<**.**001**
Secondary to RVI	232 (47.3)	154 (74.8)	78 (27.5)	**<**.**001**
Length of stay, d, median (IQR)^j^	5 (3–9)	7 (4–12)	3 (2–5)	**<**.**001**
ICU admission	38 (7.8)	36 (17.5)	2 (0.7)	**<.001**
Oxygen requirement (maximal)				
None	332 (67.8)	74 (35.8)	258 (90.8)	**<**.**001**
Nasal cannula	98 (20.0)	72 (35.0)	26 (9.2)	
Face mask	8 (1.6)	8 (3.9)	0 (0.0)	
HFNC	20 (4.1)	20 (9.7)	0 (0.0)	
BiPAP	15 (3.1)	15 (7.3)	0 (0.0)	
Mechanical ventilation	17 (3.5)	17 (8.3)	0 (0.0)	
Follow-up duration, d, median (IQR)	90 (90–90)	90 (90–90)	90 (90–90)	**<.001**
30-d all-cause mortality	20 (4.1)	20 (9.7)	0 (0.0)	**<.001**
30-d RVI-related mortality	19 (3.9)	19 (9.2)	0 (0.0)	**<.001**
90-d all-cause mortality	32 (6.5)	27 (13.1)	5 (1.8)	**<**.**001**

The difference in follow-up duration is more clearly illustrated when presented as the mean ± SD rather than the median and IQR. In the URI group, the mean duration was 85.4 ± 18.5 years, compared to 78.2 ± 26.5 years in the LRI group. *P* values less than .05 are indicated in bold format.

Abbreviations: BiPAP, bilevel positive airway pressure; CAR-T, chimeric antigen receptor T-cell; COVID-19, coronavirus disease 2019; HCT, hematopoietic stem cell transplantation; HFNC, high-flow nasal cannula; HM, hematologic malignancy; ICU, intensive care unit; IQR, interquartile range; IVIG, intravenous immunoglobulin; LRI, lower respiratory tract infection; RSV, respiratory syncytial virus; RVI, respiratory virus infection; SD, standard deviation; URI, upper respiratory tract infection.

^a^Data are presented as No. (%) unless otherwise specified.

^b^n = 489.

^c^n = 294.

^d^n = 295.

^e^Viral coinfections included rhinovirus (n = 34), seasonal human coronavirus (non–severe acute respiratory syndrome coronavirus 2 [SARS-CoV-2]) (n = 31), SARS-CoV-2 (n = 4), parainfluenza (n = 10), human metapneumovirus (n = 7), adenovirus (n = 1), and cytomegalovirus pneumonitis (n = 3).

^f^n = 462.

^g^n = 204.

^h^n = 258.

^i^Three RSV-infected patients were treated empirically with oseltamivir before the respiratory virus panel results were received.

^j^n = 280.

Patients with RSV infections had a total of 234 viral episodes, including 2 episodes with concurrent IFV infection. Ribavirin was administered to 142 (60.7%) patients, mainly using the oral formulation (97.9%), for a median duration of 6.5 days. Among the 142 patients treated with ribavirin, 57 (40.1%) presented with URI, and 7 (12.3%) progressed to LRI. However, among untreated patients, 4 of 74 (5.4%) patients with URI progressed to LRI (*P* = .208).

Patients with IFV infections had a total of 258 episodes, including 2 episodes with concurrent RSV infection, as mentioned above. Oseltamivir was administered to 242 (93.8%) patients for a median duration of 5 days. In 172 (66.7%) episodes of URI at presentation, 160 (93.0%) were treated with oseltamivir, and only 8 (4.7%) progressed to LRI within 30 days of follow-up. The timing of oseltamivir within or beyond 48 hours from symptom onset in patients with URI was associated with 6 (8.0%) versus 2 (2.4%) progressions to LRI, respectively (*P* = .148). All-cause mortality at day 30 was also similar between the 2 groups (3.6% vs 4.5%, respectively; *P* = .759). Of 58 (22.7%) patients with IFV infections who received the IFV vaccine during the relevant season, 15 (25.9%) had LRI compared to 77 (38.9%) of the nonvaccinated patients (*P* = .069).

Table 2 compares the characteristics and clinical outcomes of patients with HM stratified by virus (RSV/IFV). When compared to patients with IFV infections, more patients with RSV infections were female, had active malignancy at diagnosis, presented with or progressed to LRI, and had nosocomial infection.

**Table 2.** Baseline Characteristics and Clinical Outcomes Following Viral Infection, by Pathogen (n = 488)

Variable^a^	RSV (n = 232)	Influenza (n = 256)	*P* Value
Demographics			
Age at RVI diagnosis, y, mean ± SD	62.1 ± 14.4	61.3 ± 13.3	.514
Sex			
Female	113 (48.7)	102 (39.8)	.049
Male	119 (51.3)	154 (60.2)	
Race/ethnicity			
Non-Hispanic White	126 (54.3)	150 (58.6)	.170
Hispanic	47 (20.3)	41 (16.0)	
Black	41 (17.7)	51 (19.9)	
Asian	14 (6.0)	14 (5.5)	
Other	4 (1.7)	0 (0.0)	
Smoking status^b^			
Never	149 (64.2)	163 (63.9)	.656
Former	78 (33.6)	83 (32.6)	
Current	5 (2.2)	9 (3.5)	
Influenza vaccination (current season)	72 (31.0)	58 (22.7)	.037
HM characteristics			
Active malignancy at RVI diagnosis	176 (75.9)	173 (67.6)	.043
HM diagnosis			
Hodgkin lymphoma	14 (6.0)	17 (6.6)	.152
Non-Hodgkin lymphoma	67 (28.9)	94 (36.7)	
Multiple myeloma	151 (65.1)	145 (56.7)	
Active antineoplastic treatment at RVI diagnosis	188 (81.0)	195 (76.2)	.192
Steroid use within 30 d of RVI diagnosis (mg prednisone equivalent)			
Any	143 (61.6)	151 (59.0)	.550
30-d cumulative steroid dosage, median (IQR)^c^	600 (267–1067)	525 (220–1005)	.086
Peak dose of ≤1 mg/kg/d^d^	38 (26.6)	47 (31.3)	.369
Peak dose of >1 mg/kg/d^d^	105 (73.4)	103 (68.7)	
Previous chest radiotherapy	49 (21.1)	47 (18.4)	.443
History of HCT			
None	116 (50.0)	148 (57.8)	.195
Autologous	110 (47.4)	104 (40.6)	
Allogeneic	6 (2.6)	4 (1.6)	
History of CAR-T therapy	19 (8.2)	17 (6.6)	.513
RVI clinical course			
Site of infection at presentation			
URI	131 (56.5)	172 (67.2)	.015
LRI	101 (43.5)	84 (32.8)	
Progression to LRI (among URI)^e^	11 (8.4)	8 (4.7)	.183
Total LRI (presentation and progression)	112 (48.3)	92 (35.9)	.006
LRI type^f^			
Probable	94 (83.9)	75 (81.5)	.650
Laboratory confirmed	18 (16.1)	17 (18.5)	
Year of infection			
2016	20 (8.6)	25 (9.8)	<.001
2017	38 (16.4)	60 (23.4)	
2018	43 (18.4)	28 (10.9)	
2019	52 (22.4)	63 (24.5)	
2020	11 (4.7)	42 (16.4)	
2021	36 (15.5)	0 (0.0)	
2022	32 (13.8)	38 (14.8)	
Time period of infection			
Pre-COVID-19 era (Jan 2016–Feb 2020)	161 (69.4)	213 (83.2)	<.001
COVID-19 era (Mar 2020–Dec 2022)	71 (30.6)	43 (16.8)	
RVI symptoms			
Cough	200 (86.2)	226 (88.3)	.492
Fever	91 (39.2)	158 (61.7)	<.001
Shortness of breath	76 (32.8)	84 (32.8)	.990
Rhinorrhea	81 (34.9)	66 (25.8)	.028
Nasal congestion	75 (32.3)	76 (29.7)	.529
Fatigue	79 (34.1)	70 (27.3)	.108
Sore throat	36 (15.5)	37 (14.5)	.742
Chills	33 (14.2)	48 (18.8)	.180
Headache	19 (8.2)	35 (13.7)	.054
Nausea/vomiting	16 (6.9)	39 (15.2)	.004
Myalgia	14 (6.0)	32 (12.5)	.015
Diarrhea	12 (5.2)	26 (10.2)	.040
Chest pain	13 (5.6)	20 (7.8)	.332
Arthralgia	2 (0.9)	12 (4.7)	.011
Respiratory viral coinfection (during ±2 wk)			
Any	45 (19.4)	40 (15.6)	.273
Rhinovirus	21 (9.1)	13 (5.1)	.085
Coronavirus (non-SARS-CoV-2)	16 (6.9)	15 (5.9)	.639
SARS-CoV-2	2 (0.9)	2 (0.8)	.921
Parainfluenza	3 (1.3)	7 (2.7)	.345
Human metapneumovirus	4 (1.7)	3 (1.2)	.608
Adenovirus	1 (0.4)	0 (0.0)	.293
Cytomegalovirus (positive BAL)	2 (0.9)	1 (0.4)	.607
Nosocomial infection	16 (6.9)	2 (0.8)	<.001
Bronchoscopy	18 (7.8)	23 (9.0)	.626
Lymphopenia (<200 cells/mL)	29 (12.5)	31 (12.1)	.896
Neutropenia (<500 cells/mL)	14 (6.0)	13 (5.1)	.644
Lymphopenia and neutropenia	11 (4.7)	6 (2.3)	.149
Elevated creatinine (≥1.2 mg/dL)	67 (28.9)	78 (30.5)	.701
RVI outcomes			
Hospital admission			
Any	129 (55.6)	149 (58.2)	.562
Secondary to RVI	105 (45.3)	127 (49.6)	.336
Length of stay, d, median (IQR)^g^	5 (3–8)	5 (3–9)	.732
ICU admission	20 (8.6)	18 (7.0)	.513
Oxygen requirement (maximal)			
None	149 (64.3)	183 (71.5)	.119
Nasal cannula	53 (22.8)	44 (17.2)	
Face mask	6 (2.6)	2 (0.8)	
HFNC	8 (3.4)	11 (4.3)	
BiPAP	5 (2.2)	10 (3.9)	
Mechanical ventilation	11 (4.7)	6 (2.3)	
Antiviral timing from symptom onset			
No treatment	87 (37.5)	13 (5.1)	<.001
Within 48 h	29 (12.5)	108 (42.2)	
After 48 h	116 (50.0)	135 (52.7)	
Antiviral therapy			
Any therapy	145 (62.5)	243 (94.9)	<.001
Ribavirin	140 (60.3)	0 (0.0)	<.001
Oseltamivir	3 (1.3)	240 (93.8)	<.001
IVIG	50 (21.6)	16 (6.3)	<.001
Follow-up duration, d, median (IQR)	90 (90–90)	90 (90–90)	.819
30-d all-cause mortality	10 (4.3)	10 (3.9)	.822
30-d RVI-related mortality	9 (3.9)	10 (3.9)	.988
90-d all-cause mortality	15 (6.5)	17 (6.6)	.938

Two patients had both respiratory syncytial virus and influenza.

Abbreviations: BAL, bronchoalveolar lavage; BiPAP, bilevel positive airway pressure; CAR-T, chimeric antigen receptor T-cell; COVID-19, coronavirus disease 2019; HCT, hematopoietic stem cell transplantation; HFNC, high-flow nasal cannula; HM, hematologic malignancy; ICU, intensive care unit; IQR, interquartile range; IVIG, intravenous immunoglobulin; LRI, lower respiratory tract infection; RSV, respiratory syncytial virus; RVI, respiratory virus infection; SARS-CoV-2, severe acute respiratory syndrome coronavirus 2; SD, standard deviation; URI, upper respiratory tract infection.

^a^Data are presented as No. (%) unless otherwise specified.

^b^n = 487.

^c^n = 292.

^d^n = 293.

^e^n = 303.

^f^n = 204.

^g^n = 278.

### Risk Factors for LRI and Mortality

In a multivariable analysis, factors independently associated with LRI were RSV infection (vs IFV; adjusted odds ratio [aOR], 1.77 [95% confidence interval {CI}, 1.20–2.61]), current/former smoking (aOR, 1.64 [95% CI, 1.10–2.45]), recent steroid exposure (aOR, 2.14 [95% CI, 1.43–3.22]), lymphopenia (aOR, 3.82 [95% CI, 2.02–7.22]), and elevated creatinine level (≥1.2 mg/dL) at presentation (aOR, 2.07 [95% CI, 1.36–3.15]) (Table 3).

**Table 3.** Multivariable Analysis (Logistic Regression) of Risk Factors for Lower Respiratory Tract Infection and 30- and 90-Day All-Cause Mortality

Independent Predictor	aOR	(95% CI)	*P* Value
LRI			
Smoking (former or current)	1.64	(1.10–2.45)	.015
Lymphopenia (<200 cells/mL)	3.82	(2.02–7.22)	<.0001
Elevated creatinine (≥1.2 mg/dL)	2.07	(1.36–3.15)	.0007
Steroid use (within 30 d of RVI diagnosis)	2.14	(1.43–3.22)	.0002
Pathogen^a^			
RSV	1.77	(1.20–2.61)	.004
Influenza	Reference		
30-day all-cause mortality			
Smoking (former or current)	2.97	(1.02–8.63)	.046
Lymphopenia (<200 cells/mL)	20.12	(6.79–59.60)	<.0001
Type of cancer			
Multiple myeloma	4.08	(1.03–16.15)	.045
Lymphoma	Reference		
Nosocomial infection	12.80	(3.10–52.76)	.0004
90-d all-cause mortality			
Smoking (former or current)	2.48	(1.10–5.59)	.029
Lymphopenia (<200 cells/mL)	9.05	(3.96–20.66)	<.0001
Site of infection			
LRI	5.06	(1.83–13.95)	.002
URI	Reference		

Abbreviations: aOR, adjusted odds ratio; CI, confidence interval; LRI, lower respiratory tract infection; RSV, respiratory syncytial virus; RVI, respiratory tract infection; URI, upper respiratory tract infection.

^a^Two patients with RSV and influenza infections were excluded from the analysis.

**Figure 1.**
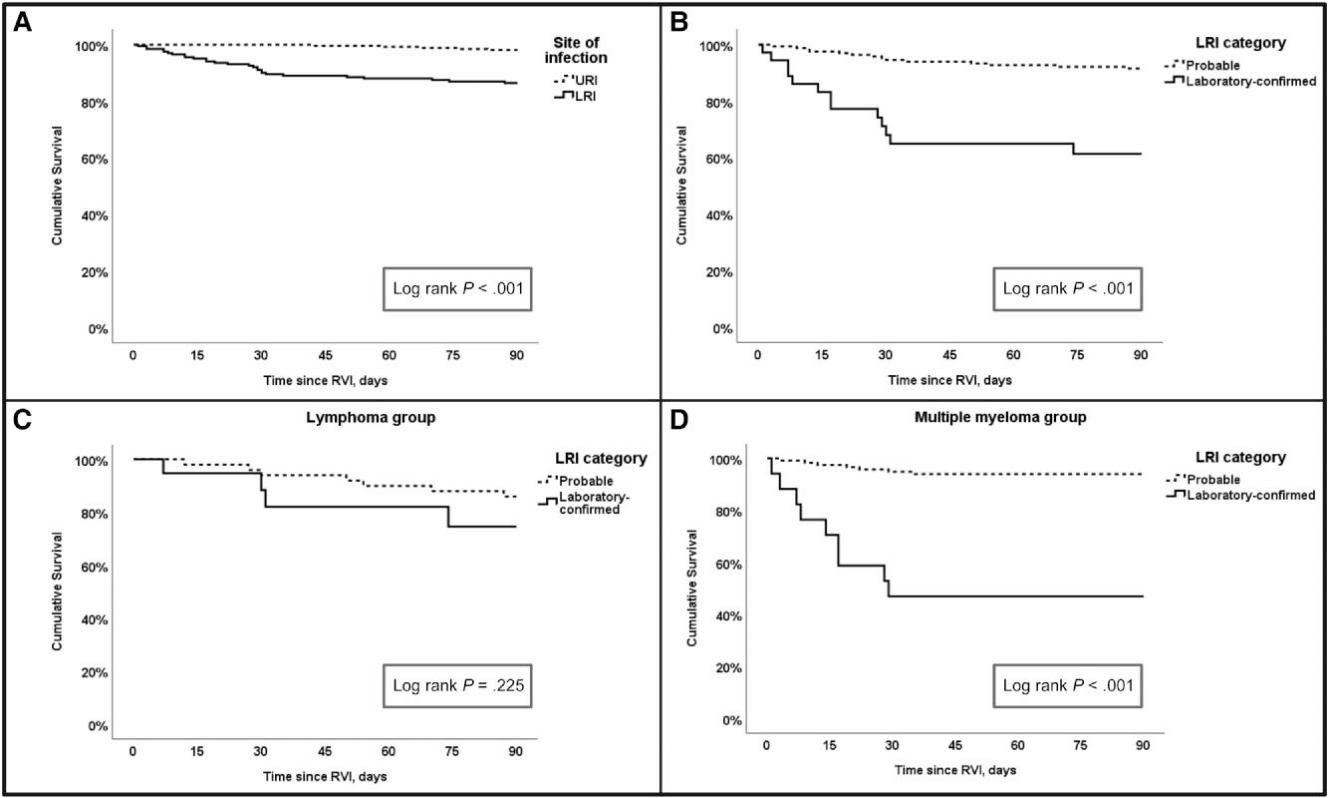
Ninety-day survival curves. *A*, Ninety-day survival according to the site of infection (within all population, n = 490). *B*, Ninety-day survival according to lower respiratory tract infection (LRI) diagnosis category (within all LRI patients, n = 206). *C*, Ninety-day survival according to the LRI diagnosis category in patients with lymphoma (n = 71). *D*, Ninety-day survival according to the LRI diagnosis category in patients with multiple myeloma (n = 135). Abbreviations: LRI, lower respiratory tract infection; RVI, respiratory viral infection; URI, upper respiratory tract infection. Created in BioRender. Shafat, T. (2025): https://BioRender.com/e13e695.

Twenty patients, 10 with RSV and 10 with IFV, died by day 30, accounting for a 4.1% all-cause mortality rate; 19 of the 20 deaths were RVI-related. The 90-day all-cause mortality rate was 6.5% (32/490). The comparisons of the characteristics of 30- and 90-day survivors and nonsurvivors are presented in Supplementary Tables 2 and 3, respectively. Notably, nineteen (95.0%) of the 20 patients who died by day 30 presented with LRI, while 1 patient experienced progression to LRI during follow-up. In a multivariable analysis, baseline MM diagnosis (vs lymphoma; aOR, 4.08 [95% CI, 1.03–16.15]), current/former smoking (aOR, 2.97 [95% CI, 1.02–8.63]), nosocomial infection (aOR, 12.8 [95% CI, 3.10–52.76]), and lymphopenia (aOR, 20.12 [95% CI, 6.79–59.60]) were independently associated with 30-day all-cause mortality (Table 3). Thirty-two patients died by day 90 (Supplementary Table 2). In a multivariable analysis, independent risk factors of the 90-day mortality were current/former smoking (aOR, 2.48 [95% CI, 1.10–5.59]), lymphopenia at presentation (aOR, 9.05 [95% CI, 3.96–20.66]), and LRI (vs URI; aOR, 5.06 [95% CI, 1.83–13.95]) (Table 3). In Kaplan-Meier survival analysis, patients with LRI (vs URI), patients with laboratory-confirmed LRI (vs probable LRI), and patients with MM and laboratory-confirmed LRI (vs patients with MM and probable LRI) were all associated with lower 90-day survival (all log-rank *P* < .001) (Figure 1  *A–D*).

Table 4 describes patients by their underlying malignancy. When compared to patients with lymphoma, more patients with MM were older, were Black, had active malignancy at RVI diagnosis, were on steroids, received autologous transplantation, were admitted for RVIs, required oxygen supplementation, and presented with or progressed to LRI. To account for the dissimilarities between patients with lymphoma and MM, separate multivariable analyses for each group were performed. In patients with lymphoma, the risk factors for LRI were older age, previous chest radiotherapy, RSV infection (rather than IFV), respiratory viral coinfection, and lymphopenia (Supplementary Table 4*A*). The risk factors for 90-day mortality were lymphopenia (aOR, 15.62 [95% CI, 3.86–63.18]; *P* < .001) and LRI (aOR, 13.20 [95% CI, 1.56–111.60]; *P* = .018). A 30-day mortality analysis was not performed due to the small number of events (n = 5).

**Table 4.** Baseline Characteristics and Clinical Outcomes Following Viral Infection (Influenza and Respiratory Syncytial Virus), by Baseline Hematological Malignancy

Variable^a^	Lymphoma (n = 193)	Multiple Myeloma (n = 297)	*P* Value
Demographics			
Age at RVI diagnosis, y, mean ± SD	58.3 ± 17.0	64.0 ± 10.7	**<.001**
Sex			
Female	79 (40.9)	137 (46.1)	.258
Male	114 (59.1)	160 (53.9)	
Race/ethnicity			
Non-Hispanic White	126 (65.3)	151 (50.8)	**<**.**001**
Hispanic	37 (19.2)	51 (17.2)	
Black	15 (7.8)	77 (25.9)	
Asian	13 (6.7)	16 (5.4)	
Other	2 (1.0)	2 (0.7)	
Smoking status^b^			
Never	130 (67.7)	184 (62.0)	.096
Former	54 (28.1)	107 (36.0)	
Current	8 (4.2)	6 (2.0)	
Influenza vaccination (current season)	30 (15.5)	100 (33.7)	**<.001**
HM characteristics			
Active malignancy at RVI diagnosis	112 (58.0)	239 (80.5)	**<.001**
Active antineoplastic treatment at RVI diagnosis	130 (67.4)	255 (85.9)	**<.001**
Steroid use within 30 d of RVI diagnosis (mg prednisone equivalent)			
Any	80 (41.5)	216 (72.7)	**<**.**001**
30-d cumulative steroid dosage, median (IQR)^c^	500 (160–907)	533 (267–1066)	.063
Peak dose ≤1 mg/kg/d^d^ (n = 295)	38 (47.5)	48 (22.3)	**<**.**001**
Peak dose >1 mg/kg/d^d^	42 (52.5)	167 (77.7)	
Previous chest radiotherapy	31 (16.1)	65 (21.9)	.113
History of HCT			
None	161 (83.4)	105 (35.4)	**<**.**001**
Autologous	25 (13.0)	189 (63.6)	
Allogeneic	7 (3.6)	3 (1.0)	
History of CAR-T therapy	18 (9.3)	18 (6.1)	.176
RVI clinical course			
Pathogen			
RSV	81 (42.0)	151 (50.9)	.155
Influenza	111 (57.5)	145 (48.8)	
RSV + influenza	1 (0.5)	1 (0.3)	
Respiratory viral coinfection (during ±2 wk)	37 (19.2)	48 (16.2)	.390
Site of infection at presentation			
URI	131 (67.9)	172 (57.9)	.**027**
LRI	62 (32.1)	125 (42.1)	
Progression to LRI (among URI)^e^	9 (6.9)	10 (5.8)	.707
Total LRI (presentation and progression)	71 (36.8)	135 (45.5)	.058
LRI type^f^			
Probable	52 (73.2)	118 (87.4)	.**001**
Laboratory confirmed	19 (26.8)	17 (12.6)	
Year of infection			
2016	13 (6.8)	32 (10.8)	.**029**
2017	50 (25.9)	48 (16.2)	
2018	29 (15.0)	44 (14.8)	
2019	51 (26.5)	64 (21.5)	
2020	18 (9.3)	35 (11.8)	
2021	13 (6.7)	23 (7.7)	
2022	19 (9.8)	51 (17.2)	
Time period of infection			
Pre–COVID-19 era (Jan 2016–Mar 2020)	159 (82.4)	217 (73.1)	.**017**
COVID-19 era (Mar 2020–Dec 2022)	34 (17.6)	80 (26.9)	
RVI symptoms			
Cough	164 (85.0)	264 (88.9)	.203
Fever	97 (50.3)	153 (51.5)	.786
Shortness of breath	56 (29.0)	106 (35.7)	.125
Rhinorrhea	63 (32.6)	85 (28.6)	.343
Nasal congestion	62 (32.1)	89 (30.0)	.613
Fatigue	66 (34.2)	83 (27.9)	.142
Sore throat	30 (15.5)	43 (14.5)	.746
Chills	29 (15.0)	52 (17.5)	.470
Headache	22 (11.4)	32 (10.8)	.829
Nausea/vomiting	23 (11.9)	32 (10.8)	.695
Myalgia	22 (11.4)	24 (8.1)	.219
Diarrhea	15 (7.8)	23 (7.7)	.991
Chest pain	14 (7.3)	19 (6.4)	.712
Arthralgia	7 (3.6)	7 (2.4)	.410
Hypoxia at presentation (≤92%) in room air^g^	17 (9.3)^h^	32 (11.4)^i^	.476
Nosocomial infection	10 (5.2)	10 (3.4)	.321
Lymphopenia (<200 cells/mL)	21 (10.9)	39 (13.1)	.458
Neutropenia (<500 cells/mL)	17 (8.8)	11 (3.7)	.017
Lymphopenia and neutropenia	8 (4.1)	9 (3.0)	.510
Elevated creatinine (≥1.2 mg/dL)	37 (19.2)	109 (36.7)	**<.001**
RVI outcomes			
Hospital admission			
Any	100 (51.8)	180 (60.6)	.055
Secondary to RVI	77 (39.9)	155 (52.2)	.**008**
Length of stay, d, median (IQR)^j^	6 (3–9)	5 (3–9)	.**429**
ICU admission	19 (9.8)	19 (6.4)	.163
Oxygen requirement (maximal)			
None	143 (74.1)	189 (63.6)	.**031**
Nasal cannula	27 (14.0)	71 (23.9)	
Face mask	2 (1.0)	6 (2.0)	
HFNC	9 (4.7)	11 (3.7)	
BiPAP	3 (1.6)	12 (4.0)	
Mechanical ventilation	9 (4.7)	8 (2.7)	
Antiviral timing from symptom onset			
No treatment	48 (24.9)	52 (17.5)	.073
Within 48 h	57 (29.5)	82 (27.6)	
After 48 h	88 (45.6)	163 (54.9)	
Antiviral therapy			
Any therapy	145 (75.1)	245 (82.5)	.**048**
Ribavirin	42 (21.8)	100 (33.7)	.**005**
Oseltamivir	101 (52.3)	144 (48.5)	.405
IVIG	15 (7.8)	52 (17.5)	.**002**
Bronchoscopy	21 (10.9)	21 (7.1)	.141
Follow-up duration, d, median (IQR)	90 (90–90)	90 (90–90)	.215
30-d all-cause mortality	5 (2.6)	15 (5.1)	.179
30-d RVI-related mortality	4 (2.1)	15 (5.1)	.095
90-d all-cause mortality	12 (6.2)	20 (6.7)	.821

*P* values less than .05 are indicated in bold format.

Abbreviations: BiPAP, bilevel positive airway pressure; CAR-T, chimeric antigen receptor T-cell; COVID-19, coronavirus disease 2019; HCT, hematopoietic stem cell transplantation; HFNC, high-flow nasal cannula; ICU, intensive care unit; IQR, interquartile range; IVIG, intravenous immunoglobulin; LRI, lower respiratory tract infection; RSV, respiratory syncytial virus; RVI, respiratory virus infection; SD, standard deviation; URI, upper respiratory tract infection.

^a^Data are presented as No. (%) unless otherwise specified.

^b^n = 489.

^c^n = 294.

^d^n = 295.

^e^n = 303.

^f^n = 206.

^g^n = 462.

^h^n = 182.

^i^n = 280.

^j^n = 280.

In patients with MM, recent steroid exposure, lymphopenia, and elevated creatinine levels were associated with LRI (Supplementary Table 4*B*). The risk factors for 30-day mortality were lymphopenia (aOR, 13.78 [95% CI, 4.29–44.22]; *P* = .001) and nosocomial infection (aOR, 12.37 [95% CI, 2.21–69.26]; *P* = .004), whereas the risk factors for 90-day mortality were lymphopenia (aOR, 6.43 [95% CI, 2.34–17.68]; *P* < .001), LRI (aOR, 3.95 [95% CI, 1.20–13.04]; *P* = .024), and nosocomial infection (aOR, 8.12 [95% CI, 1.63–40.40]; *P* = .011) (Supplementary Table 4*B*).

### IFV and RSV Seasonality

**Figure 2.**
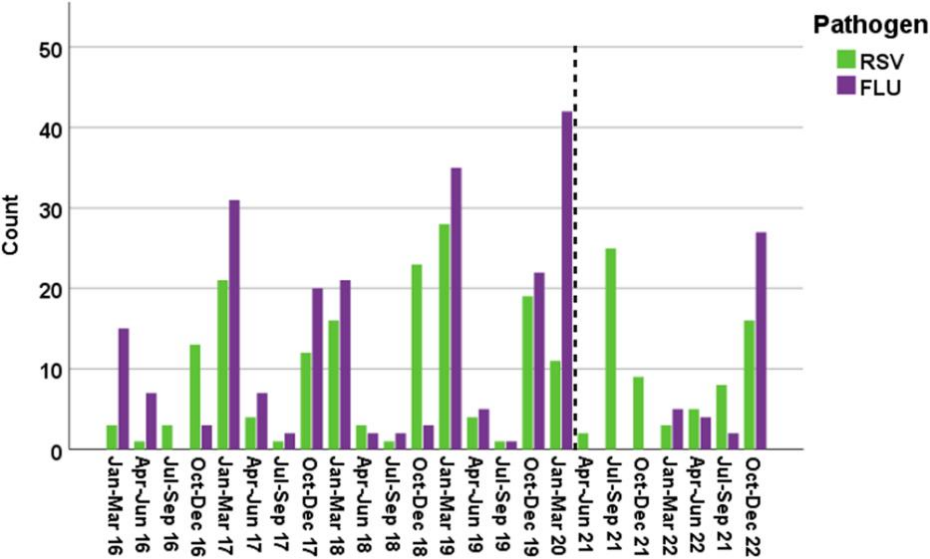
Respiratory viral infections per quarter stratified by viral pathogen. The dashed line represents the beginning of the coronavirus disease 2019 pandemic in the United States (March 2020). Abbreviations: IFV, influenza virus; RSV, respiratory syncytial virus. Created in BioRender. Shafat, T. (2025): https://BioRender.com/e53z718.

**Figure 3.**
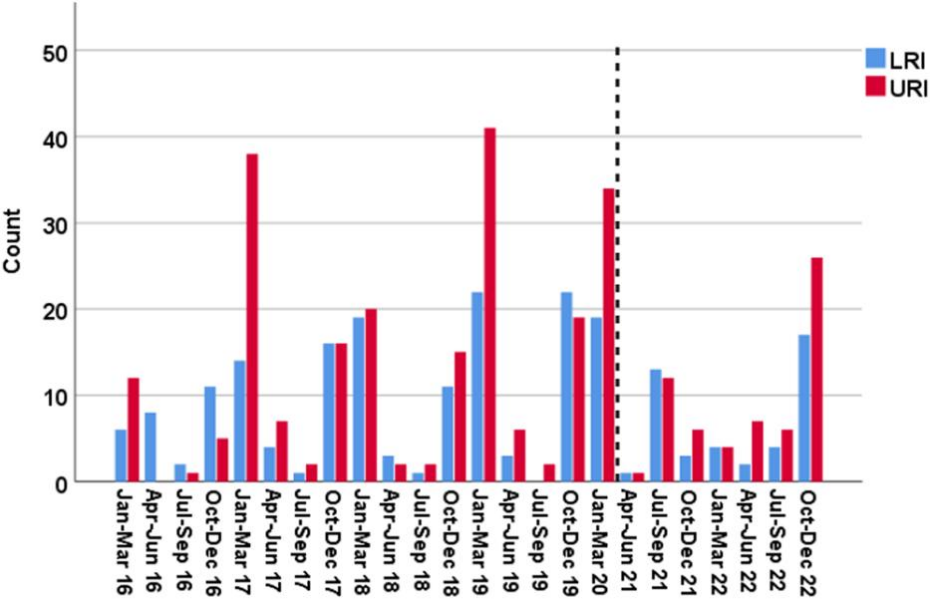
Respiratory viral infections per quarter, stratified by site of infection. The dashed line represents the beginning of the coronavirus disease 2019 pandemic in the United States (March 2020). The lower respiratory tract infection (LRI) group includes patients who presented with LRI or had progression from upper respiratory tract infection (URI) to LRI. Created in BioRender. Shafat, T. (2025): https://BioRender.com/g12o358.

This study included 7 years of RVI episodes; 77% occurred before the COVID-19 pandemic (January 2016–February 2020) (Figures 2 and 3), when the seasonality of RVIs was predictable, with cases peaking during the winter months (October–March). From March 2020, when the COVID-19 pandemic started in the United States, until June 2021, there were no documented RSV/IFV infections in our cohort of patients with lymphoma and MM, and until February 2022, there were no IFV cases. Furthermore, since March 2020, more IFV/RSV cases have been diagnosed in patients with MM compared to patients with lymphoma (26.9% vs 17.6% of cases, *P* < .017) (Table 4). Total LRI and 30-day mortality rates among infected individuals were like those in the pre-COVID-19 era (42.1% vs 42.0% LRIs and 4.4% vs 4.0% 30-day mortality rates, respectively).

## DISCUSSION

We identified a high burden of IFV and RSV infections among patients with lymphoma and MM at our center from 2016 to 2022. In this cohort, we found a high rate of RVI-related LRI (42%), with an RVI-related mortality of 3.9% at 30 days. Additionally, patients were often admitted to the hospital, with 32.5% requiring oxygen supplementation and 7.8% requiring ICU admission. We identified multiple factors associated with RVI-related LRI, including smoking status, lymphopenia, elevated creatinine level, and steroid use; additionally, we determined that almost half of the RSV infections were associated with LRI. There were significant associations between smoking status, lymphopenia, type of underlying malignancy (MM), nosocomial infection, and 30-day mortality.

Patients with HM are at significant risk for RVI complications, including progression to LRI and mortality, due to multiple host-related factors, such as immunosuppressive therapy and underlying disease. Yet there are only limited data on RVIs other than COVID-19 in patients with lymphoma and MM [13, 14]. Previous studies have reported higher rates of LRI and RVI-related mortality in patients with MM than in those with lymphoma [9, 10]. This difference was previously attributed to impaired lymphocyte function, more aggressive chemotherapy, higher autologous transplant rate, and hypogammaglobulinemia in MM patients [8, 15]. In our cohort, lymphopenia and steroid exposure were associated with LRI and 30- and 90-day mortality; additionally, patients with MM were at greater 30-day mortality risk.

Prior studies in cancer patients have reported high rates of LRI and mortality associated with IFV and RSV [2, 5, 16]. In patients with HM, reported progression rates to LRI range from 20% to 60%, depending on the degree of immunosuppression, history of HCT, and antiviral treatment [3, 7, 16–18]; among patients with lymphoma and MM, LRI rates can be as high as 75% [4, 7, 9, 10]. In our study, RSV infection was more strongly associated with LRI than IFV. The most significant difference was noted in patients with lymphoma. Both viral infections had similar 30- and 90-day mortality rates, around 4.3% and 6.5% for RSV and 3.9% and 6.6% for IFV, respectively.

The higher rate of RSV LRI in our cohort may be due to the lack of effective antiviral therapy and vaccinations (until recently) to treat or prevent RSV infection complications. Neuraminidase inhibitors such as oseltamivir are widely available to treat IFV-related URI or LRI [19]. Early treatment of IFV infections with oseltamivir has been associated with decreased rates of IFV LRI in HCT recipients [20], and annual IFV vaccination among patients with HM was associated with improved outcomes [19, 21, 22]. For instance, Kumar et al found that the current-season IFV vaccination reduced LRI risk by 66% and ICU admission risk by 51% among posttransplant patients infected with IFV [23]. In patients with lymphoma, IFV vaccination resulted in a good immune response [24]. In our cohort, oseltamivir was administered in almost all influenza cases (93.8%); however, we did note a low vaccination rate (26.5%). By comparison, no approved treatment for RSV is available, and the newly approved RSV vaccines were not commercially available during the study period [25, 26]. Furthermore, RSV vaccines are recommended for older adults, and their efficacy in immunocompromised patients has yet to be determined. Ribavirin therapy for RSV infections in high-risk patients with HM has been used, but there is a lack of high-quality clinical efficacy data [4, 27, 28]. Therefore, well-designed clinical trials of antiviral agents and vaccines for immunocompromised patients infected with respiratory viruses such as IFV and RSV are needed as the burden of LRI and mortality is substantial.

IFV and RSV seasonality was constant and predictable for decades until the COVID-19 pandemic began in March 2020. The circulation of severe acute respiratory syndrome coronavirus 2 resulted in significant changes in the presence and timing of RSV and IFV circulation, as reported in numerous epidemiologic studies among the general population [29–31]. According to the Centers for Disease Control and Prevention, RSV infections peaked earlier than usual during the 2022–2023 respiratory virus season, in October and November [30], and IFV also peaked earlier, during December and January [29]. Evaluating the seasonality of those viruses is crucial for vaccination timing and diagnostic approaches.

Our study has several limitations. First, our cohort included 2 distinct subpopulations: patients with lymphoma and MM, whose diseases have different pathogenesis, antineoplastic treatment regimens, and prognosis. By analyzing the risk factors and outcomes as 1 group, we might draw conclusions that do not reflect 1 of the subpopulations. However, we elected to combine those groups because of their underlying B- and T-cell deficiencies that distinguish them from other patients, such as HCT recipients. To account for these dissimilarities, we performed a separate multivariable analysis for each group. Second, few patients in our cohort experienced progression from URI to LRI during the study period (rather than being diagnosed with LRI at presentation); thus, a multivariate analysis to identify specific risk factors for progression to LRI was not attempted (Supplementary Table 1). Third, our cohort included only patients tested at our institution, and we probably missed infected patients diagnosed in outside facilities or mildly symptomatic patients who were not tested at all. Furthermore, the study describes a single-center experience that could affect the generalizability of the results. However, to our knowledge, this is the largest cohort that includes patients with lymphoma and MM and RVIs, and most of the results align with data from previous cohorts. Last, the study's retrospective nature affects the ability to account for potential confounders. We tried to adjust for the most clinically important ones using multivariable analyses.

In summary, our study describes the high burden of IFV and RSV infections in patients with lymphoma and MM. We also identified unique risk factors associated with LRI and mortality in this population. Of importance, we note that RSV may be associated with worse outcomes in patients with lymphoma and MM compared to IFV. This highlights the need for prospective studies to measure the rates of RSV- or IFV-related complications in patients with HM, investigating the effects of vaccination and antiviral therapy on significant clinical outcomes.

## Supplementary Material

ofaf127_Supplementary_Data

## Supplementary Data

Supplementary materials are available at *Open Forum Infectious Diseases* online. Consisting of data provided by the authors to benefit the reader, the posted materials are not copyedited and are the sole responsibility of the authors, so questions or comments should be addressed to the corresponding author.

## Notes

***Author contributions.*** D. R.-M., D. V.-C., M. V. B., and R. F. C. conceived and designed the study. D. R.-M. and T. S. extracted, collected, and validated the data. Y. J. and T. S. performed the analysis. Y. J. supervised the analysis. D. R.-M., D. V.-C., M. V. B., T. S., F. K., and R. F. C. interpreted the data. D. R.-M. and T. S. wrote the original draft. F. K., Y. J., A. S., E. A. H., D. V.-C., S. A., M. B. and R. F. C. reviewed and edited the manuscript. R. F. C. supervised the work. All authors reviewed the manuscript and approved the final version of the manuscript.

***Acknowledgments.*** The authors thank Amy Ninetto of the Research Medical Library at The University of Texas MD Anderson Cancer Center for editing the manuscript.
