# Supplementary material for: Outcomes and Risk Factors for Influenza and Respiratory Syncytial Virus Lower Respiratory Tract Infections and Mortality in Patients With Lymphoma or Multiple Myeloma: A 7-Year Retrospective Cohort Study
Source: Open Forum Infect Dis. 2025 Mar 4;12(4):ofaf127. doi: 10.1093/ofid/ofaf127 (PMC11961405; doi:10.1093/ofid/ofaf127)
Supplement: ofaf127_Supplementary_Data [file ofaf127_supplementary_data.docx]

Supplementary materials:

Table S1: Baseline characteristics and clinical outcomes of patients with hematologic malignancy and respiratory viral infection who presented with upper respiratory tract infection.

| **Variable^a^** | | **Total (n=303)** | **Progression to LRI (n=19)** | **No progression to LRI (n=284)** | ***P* value** |
| --- | --- | --- | --- | --- | --- |
| **Demographics** | | | | | |
| Age at RVI diagnosis, y (mean ±SD) | | 60.7±14.1 | 69.2±13.9 | 60.2±14.0 | **0.007** |
| Sex | Female | 134 (44.2) | 8 (42.1) | 126 (44.4) | 0.848 |
|  | Male | 169 (55.8) | 11 (57.9) | 158 (55.6) |  |
| Race/ethnicity | Non-Hispanic White | 174 (57.4) | 13 (68.4) | 161 (56.7) | 0.874 |
|  | Hispanic | 51 (16.8) | 2 (10.5) | 49 (17.3) |  |
|  | Black | 55 (18.2) | 3 (15.8) | 52 (18.3) |  |
|  | Asian | 20 (6.6) | 1 (5.3) | 19 (6.7) |  |
|  | Other | 3 (1.0) | 0 (0.0) | 3 (1.0) |  |
| Smoking status | Never | 205 (67.7) | 10 (52.6) | 195 (68.7) | 0.148 |
|  | Former/current | 98 (32.3) | 9 (47.4) | 89 (31.3) |  |
| Influenza vaccination (current season) | | 83 (27.4) | 4 (21.1) | 79 (27.8) | 0.522 |
| **HM characteristics** | | | | | |
| Hematologic malignancy diagnosis | lymphoma | 131 (43.2) | 9 (47.4) | 122 (43.0) | 0.707 |
|  | Multiple myeloma | 172 (56.8) | 10 (52.6) | 162 (57.0) |  |
| Active malignancy at RVI diagnosis | | 200 (66.0) | 16 (84.2) | 184 (64.8) | 0.084 |
| Active antineoplastic treatment at RVI diagnosis | | 228 (75.2) | 17 (89.5) | 211 (74.3) | 0.175 |
| Steroid use within 30 days of RVI diagnosis (mg prednisone equivalent) | Any | 156 (51.5) | 11 (57.9) | 145 (51.1) | 0.640 |
|  | 30-day cumulative steroid dosage (median, IQR)^b^ | 533, 170-1066 | 525, 160-1067 | 533, 172-1066 | 0.917 |
|  | Peak dose of  ≤ 1 mg/kg/day^b^ | 47 (30.3) | 4 (36.4) | 43 (29.9) | 0.736 |
|  | Peak dose of  >1 mg/kg/day^b^ | 108 (69.7) | 7 (63.6) | 101 (70.1) |  |
| Previous chest radiotherapy | | 52 (17.2) | 3 (15.8) | 49 (17.3) | 1.000 |
| History of HCT | None | 171 (56.5) | 12 (63.2) | 159 (56.0) | 0.752 |
|  | Autologous | 128 (42.2) | 7 (36.8) | 121 (42.6) |  |
|  | Allogeneic | 4 (1.3) | 0 (0.0) | 4 (1.4) |  |
| History of CAR-T therapy | | 25 (8.3) | 0 (0.0) | 25 (8.8) | 0.383 |
| **RVI clinical course** | | | | | |
| Pathogen | RSV | 131 (43.2) | 11 (57.9) | 120 (42.3) | 0.183 |
|  | Influenza | 172 (56.8) | 8 (42.1) | 164 (57.7) |  |
|  | RSV + influenza | (0.0) | (0.0) | (0.0) |  |
| Respiratory viral coinfections (during ± 2 weeks) * | | 50 (16.5) | 5 (26.3) | 45 (15.8) | 0.216 |
| Year of infection | 2016 | 21 (6.9) | 3 (15.8) | 18 (6.3) | 0.125 |
|  | 2017 | 69 (22.9) | 6 (31.5) | 63 (22.3) |  |
|  | 2018 | 43 (14.2) | 4 (21.1) | 39 (13.7) |  |
|  | 2019 | 68 (22.4) | 0 (0.0) | 68 (23.9) |  |
|  | 2020 | 37 (12.2) | 3 (15.8) | 34 (12.0) |  |
|  | 2021 | 21 (6.9) | 2 (10.5) | 19 (6.7) |  |
|  | 2022 | 44 (14.5) | 1 (5.3) | 43 (15.1) |  |
| Time period of infection | Pre-COVID-19 era (1/2016-2/2020) | 232 (76.6) | 14 (73.7) | 218 (76.8) | 0.759 |
|  | COVID-19 era (3/2020-12/2022) | 71 (23.4) | 5 (26.3) | 66 (23.2) |  |
| LRI type^c^ | Probable | - | 16 (84.2) | - | - |
|  | Laboratory confirmed | - | 3 (15.8) | - |  |
| RVI symptoms | Cough | 259 (85.5) | 15 (78.9) | 244 (85.9) | 0.496 |
|  | Fever | 141 (46.5) | 7 (36.8) | 134 (47.2) | 0.382 |
|  | Shortness of breath | 71 (23.4) | 4 (21.1) | 67 (23.6) | 1.000 |
|  | Rhinorrhoea | 113 (37.3) | 5 (26.3) | 108 (38.0) | 0.307 |
|  | Nasal congestion | 105 (34.7) | 5 (26.3) | 100 (35.2) | 0.430 |
|  | Fatigue | 86 (28.4) | 6 (31.6) | 80 (28.2) | 0.750 |
|  | Sore throat | 50 (16.5) | 3 (15.8) | 47 (16.5) | 1.000 |
|  | Chills | 46 (15.2) | 2 (10.5) | 44 (15.5) | 0.749 |
|  | Headache | 35 (11.6) | 1 (5.3) | 34 (12.0) | 0.709 |
|  | Nausea/vomiting | 31 (10.2) | 1 (5.3) | 30 (10.6) | 0.705 |
|  | Myalgia | 32 (10.6) | 2 (10.5) | 30 (10.6) | 1.000 |
|  | Diarrhea | 21 (6.9) | 2 (10.5) | 19 (6.7) | 0.630 |
|  | Chest pain | 15 (5.0) | 0 (0.0) | 15 (5.3) | 0.610 |
|  | Arthralgia | 10 (3.3) | 0 (0.0) | 10 (3.5) | 1.000 |
| Hypoxia at presentation (≤92%) in room air^d^ | | 10 (3.6) | 4 (23.5) | 6 (2.3) | **0.002** |
| Nosocomial infection | | 8 (2.6) | 0 (0.0) | 8 (2.8) | 1.000 |
| Bronchoscopy | | 4 (1.3) | 3 (15.8) | 1 (0.4) | **0.001** |
| Lymphopenia (<200 cells/ml) | | 20 (6.6) | 4 (21.1) | 16 (5.6) | **0.028** |
| Neutropenia (<500 cells/ml) | | 11 (3.6) | 2 (10.5) | 9 (3.2) | 0.146 |
| Lymphopenia & neutropenia | | 5 (1.7) | 2 (10.5) | 3 (1.1) | **0.033** |
| Elevated creatinine (≥1.2 mg/dl) | | 75 (24.8) | 9 (47.4) | 66 (23.2) | **0.027** |
| Antiviral therapy | Any therapy | 221 (72.9) | 15 (78.9) | 206 (72.5) | 0.542 |
|  | Ribavirin | 57 (18.8) | 7 (36.8) | 50 (17.6) | 0.062 |
|  | Oseltamivir | 162 (53.3) | 8 (42.1) | 154 (54.2) | 0.305 |
|  | IVIG | 17 (5.6) | 4 (21.1) | 13 (4.6) | **0.016** |
| Antiviral timing from symptom onset | No treatment | 82 (27.1) | 4 (21.1) | 78 (27.5) | 0.732 |
|  | Within 48 h | 90 (29.7) | 7 (36.8) | 83 (29.2) |  |
|  | After 48 h | 131 (43.2) | 8 (42.1) | 123 (43.3) |  |
| **RVI outcomes** | | | | | |
| Hospital admission | Any | 115 (38.0) | 15 (78.9) | 100 (35.2) | **<0.001** |
|  | Secondary to RVI | 92 (30.4) | 14 (73.7) | 78 (27.5) | **<0.001** |
|  | Length of stay (median, IQR)^e^ | 4, 3-6 | 8, 6-18 | 3, 2-5 | **<0.001** |
| ICU admission | | 5 (1.7) | 3 (15.8) | 2 (0.7) | **0.002** |
| Oxygen requirement (maximal) | None | 265 (87.5) | 7 (36.8) | 258 (90.8) | **<0.001** |
|  | Nasal cannula | 31 (10.2) | 5 (26.3) | 26 (9.2) |  |
|  | Face mask | 4 (1.3) | 4 (21.1) | 0 (0.0) |  |
|  | HFNC | 2 (0.7) | 2 (10.5) | 0 (0.0) |  |
|  | Mechanical ventilation | 1 (0.3) | 1 (5.3) | 0 (0.0) |  |
| Follow-up duration, days (median, IQR) | | 90, 90-90 | 90, 90-90 | 90, 90-90 | 0.562 |
| 30-day all-cause mortality | | 1 (0.3) | 1 (5.3) | 0 (0.0) | 0.063 |
| 30-day RVI-related mortality | | 1 (0.3) | 1 (5.3) | 0 (0.0) | 0.063 |
| 90-day all-cause mortality n | | 7 (2.3) | 2 (10.5) | 5 (1.8) | 0.065 |

^a^Data are no. (%) unless otherwise specified; ^b^n=155; ^c^n=19; ^d^n=275; ^e^n= 115.

*Viral co-infections included rhinovirus (n=19), seasonal human coronavirus (non-SARS-CoV-2) (n=21), SARS-CoV-2 (n=1), parainfluenza (n=9), and human metapneumovirus (n=3).

Abbreviations: BiPAP, bilevel positive airway pressure; CAR-T, chimeric antigen receptor T-cell therapy; COVID-19, coronavirus disease 2019; HCT, hematopoietic stem cell transplantation; HFNC, high-flow nasal cannula; HM, hematologic malignancy; ICU, intensive care unit; IQR, interquartile range; IVIG, intravenous immunoglobulin; LRI, lower respiratory tract infection; RSV, respiratory syncytial virus; RVI, respiratory virus infection; SD, standard deviation; URI, upper respiratory tract infection.

**Table S2: Baseline characteristics and clinical outcomes of patients with hematologic malignancy and respiratory viral infection, by 30-day survival.**

| **Variable^a^** | | **Non-survivors (n=20)** | **Survivors**  **(n=470)** | ***P* value** |
| --- | --- | --- | --- | --- |
| **Demographics** | | | | |
| Age at RVI diagnosis, years (mean ±SD) | | 62.3±11.4 | 61.7±13.9 | 0.855 |
| Sex | Female | 8 (40.0) | 208 (44.3) | 0.707 |
|  | Male | 12 (60.0) | 262 (55.7) |  |
| Race/ethnicity | Non-Hispanic White | 14 (70.0) | 263 (56.0) | 0.587 |
|  | Hispanic | 2 (10.0) | 86 (18.3) |  |
|  | Black | 4 (20.0) | 88 (18.7) |  |
|  | Asian | 0 (0.0) | 29 (6.2) |  |
|  | Other | 0 (0.0) | 4 (0.9) |  |
| Smoking status^b^ | Never | 9 (47.4) | 305 (64.9) | 0.152 |
|  | Former | 10 (52.6) | 151 (32.1) |  |
|  | Current | 0 (0.0) | 14 (3.0) |  |
| Influenza vaccination (current season) | | 3 (15.0) | 127 (27.0) | 0.233 |
| **HM characteristics** | | | | |
| Hematologic malignancy diagnosis | Hodgkin lymphoma | 1 (5.0) | 30 (6.4) | 0.397 |
|  | Non-Hodgkin lymphoma | 4 (20.0) | 158 (33.6) |  |
|  | Multiple myeloma | 15 (75.0) | 282 (60.0) |  |
| Active malignancy at RVI diagnosis | | 20 (100.0) | 331 (70.4) | **0.004** |
| Active antineoplastic treatment at RVI diagnosis | | 18 (90.0) | 367 (78.1) | 0.272 |
| Steroid use within 30 days of RVI diagnosis (mg prednisone equivalent) | Any | 19 (95.0) | 277 (58.9) | **0.001** |
|  | 30-day cumulative steroid dosage (median, IQR)^c^ | 533, 266-1300 | 533, 240-1066 | 0.428 |
|  | Peak dose of  ≤1 mg/kg/day^d^ | 5 (26.3) | 81 (29.3) | 0.778 |
|  | Peak dose of  >1 mg/kg/day^d^ | 14 (73.7) | 195 (70.7) |  |
| Previous chest radiotherapy | | 6 (30.0) | 90 (19.1) | 0.249 |
| History of HCT | None | 12 (60.0) | 254 (54.0) | 0.502 |
|  | Autologous | 7 (35.0) | 207 (44.0) |  |
|  | Allogeneic | 1 (5.0) | 9 (1.9) |  |
| History of CAR-T therapy | | 1 (5.0) | 35 (7.4) | 1.000 |
| **RVI clinical course** | | | | |
| Pathogen | RSV | 10 (50.0) | 222 (47.2) | 0.934 |
|  | Influenza | 10 (50.0) | 246 (52.3) |  |
|  | RSV + influenza | 0 (0.0) | 2 (0.4) |  |
| Respiratory viral coinfection (during ± 2 weeks) | | 4 (20.0) | 81 (17.2) | 0.749 |
| Year of infection | 2016 | 3 (15.0) | 42 (8.9) | 0.417 |
|  | 2017 | 2 (10.0) | 96 (20.4) |  |
|  | 2018 | 4 (20.0) | 69 (14.7) |  |
|  | 2019 | 5 (25.0) | 110 (23.4) |  |
|  | 2020 | 1 (5.0) | 52 (11.1) |  |
|  | 2021 | 0 (0.0) | 36 (7.7) |  |
|  | 2022 | 5 (25.0) | 65 (13.8) |  |
| Time period of infection | Pre COVID-19 era (1/2016-2/2020) | 15 (75.0) | 361 (76.8) | 0.792 |
|  | COVID-19 era (2/2020-12/2022) | 5 (25.0) | 109 (23.2) |  |
| Site of infection at presentation | URI | 1 (5.0) | 302 (64.3) | **<0.001** |
|  | LRI | 19 (95.0) | 168 (35.7) |  |
| Progression to LRI (among URI)^e^ | | 1 (100.0) | 18 (6.0) | **<0.001** |
| Total LRI (presentation and progression) | | 20 (100.0) | 186 (39.6) | **<0.001** |
| LRI type^f^ | Probable | 9 (45.0) | 161 (86.6) | **<0.001** |
|  | Laboratory confirmed | 11 (55.0) | 25 (13.4) |  |
| RVI symptoms | Cough | 14 (70.0) | 414 (88.1) | **0.030** |
|  | Fever | 6 (30.0) | 244 (51.9) | 0.055 |
|  | Shortness of breath | 8 (40.0) | 154 (32.8) | 0.501 |
|  | Rhinorrhoea | 0 (0.0) | 148 (31.5) | **0.003** |
|  | Nasal congestion | 2 (10.0) | 149 (31.7) | **0.047** |
|  | Fatigue | 13 (65.0) | 136 (28.9) | **0.001** |
|  | Sore throat | 1 (5.0) | 72 (15.3) | 0.335 |
|  | Chills | 1 (5.0) | 80 (17.0) | 0.223 |
|  | Headache | 1 (5.0) | 53 (11.3) | 0.713 |
|  | Nausea/vomiting | 3 (15.0) | 52 (11.1) | 0.482 |
|  | Myalgia | 0 (0.0) | 46 (9.8) | 0.241 |
|  | Diarrhoea | 0 (0.0) | 38 (8.1) | 0.389 |
|  | Chest pain | 3 (15.0) | 30 (6.4) | 0.145 |
|  | Arthralgia | 0 (0.0) | 14 (3.0) | 1.000 |
| Hypoxia at presentation (≤92%) in room air^g^ | | 11 (57.9)^h^ | 38 (8.6)^i^ | **<0.001** |
| Nosocomial infection | | 5 (25.0) | 15 (3.2) | **0.001** |
| Bronchoscopy | | 13 (65.0) | 29 (6.2) | **<0.001** |
| Lymphopenia (<200 cells/ml) | | 14 (70.0) | 46 (9.8) | **<0.001** |
| Neutropenia (<500 cells/ml) | | 4 (20.0) | 24 (5.1) | **0.022** |
| Lymphopenia & neutropenia | | 4 (20.0) | 13 (2.8) | **0.003** |
| Elevated creatinine (≥1.2 mg/dl) | | 10 (50.0) | 136 (28.9) | **0.044** |
| Antiviral therapy n (%) | Any therapy | 20 (100.0) | 370 (78.7) | **0.019** |
|  | Ribavirin | 10 (50.0) | 132 (28.1) | **0.034** |
|  | Oseltamivir | 11 (55.0) | 234 (49.8) | 0.648 |
|  | IVIG | 6 (30.0) | 61 (13.0) | **0.042** |
| Anti-viral timing from symptoms onset | No treatment | 0 (0.0) | 100 (21.3) | 0.058 |
|  | Within 48 hours | 6 (30.0) | 133 (28.3) |  |
|  | After 48 hours | 14 (70.0) | 237 (50.4) |  |
| **RVI outcomes** | | | | |
| Hospital admission | Any | 20 (100.0) | 260 (55.3) | **<0.001** |
|  | Secondary to RVI | 12 (60.0) | 220 (46.8) | 0.247 |
|  | Length of stay (median, IQR)^j^ | 16, 9-28 | 5, 3-8 | <0.001 |
| ICU admission | | 14 (70.0) | 24 (5.1) | **<0.001** |
| Oxygen requirement (maximal) | None | 1 (5.0) | 331 (70.4) | **<0.001** |
|  | Nasal cannula | 2 (10.0) | 96 (20.4) |  |
|  | Face mask | 0 (0.0) | 8 (1.7) |  |
|  | HFNC | 4 (20.0) | 16 (3.4) |  |
|  | BiPAP | 5 (25.0) | 10 (2.1) |  |
|  | Mechanical ventilation | 8 (40.0) | 9 (1.9) |  |
| Follow-up duration, days (median, IQR) | | 15, 7-27 | 90, 90-90 | **<0.001** |

^a^Data are no. (%) unless otherwise specified; ^b^n=489; ^c^n=294; ^d^n=295; ^e^n=303; ^f^n=206; ^g^n=462; ^h^n=19; ^i^n=443, ^j^n=280.

Abbreviations: BiPAP, bilevel positive airway pressure; CAR-T, chimeric antigen receptor T-cell therapy; COVID-19, coronavirus disease 2019; HCT, hematopoietic stem cell transplantation; HFNC, high-flow nasal cannula; HM, hematologic malignancy; ICU, intensive care unit; IQR, interquartile range; IVIG, intravenous immunoglobulin; LRI, lower respiratory tract infection; RSV, respiratory syncytial virus; RVI, respiratory virus infection; SD, standard deviation; URI, upper respiratory tract infection.

**Table S3: Baseline characteristics and clinical outcomes among patients with hematologic malignancy and respiratory viral infection, by 90-day survival.**

| **Variable^a^** | | **Non-survivors (n=32)** | **Survivors**  **(n=458)** | ***P* value** |
| --- | --- | --- | --- | --- |
| **Demographics** | | | | |
| Age at RVI diagnosis, years (mean ±SD) | | 63.2±11.1 | 61.7±14.0 | 0.533 |
| Sex | Female | 13 (40.6) | 203 (44.3) | 0.648 |
|  | Male | 19 (59.4) | 255 (55.7) |  |
| Race/ethnicity | Non-Hispanic White | 22 (68.8) | 255 (55.7) | 0.310 |
|  | Hispanic | 3 (9.4) | 85 (18.6) |  |
|  | Black | 7 (21.9) | 85 (18.6) |  |
|  | Asian | 0 (0.0) | 29 (6.3) |  |
|  | Other | 0 (0.0) | 4 (0.9) |  |
| Smoking status^b^ | Never | 14 (45.2) | 300 (65.5) | 0.067 |
|  | Former | 16 (51.6) | 145 (31.7) |  |
|  | Current | 1 (3.2) | 13 (2.8) |  |
| Influenza vaccination (current season) | | 4 (12.5) | 126 (27.5) | 0.063 |
| **HM characteristics** | | | | |
| Hematologic malignancy diagnosis | Hodgkin lymphoma | 1 (3.1) | 30 (6.6) | 0.744 |
|  | Non-Hodgkin lymphoma | 11 (34.4) | 151 (33.0) |  |
|  | Multiple myeloma | 20 (62.5) | 277 (60.4) |  |
| Active malignancy at RVI diagnosis | | 31 (96.9) | 320 (69.9) | **0.001** |
| Active antineoplastic treatment at RVI diagnosis | | 30 (93.8) | 355 (77.5) | **0.030** |
| Steroid use within 30 days of RVI diagnosis (mg prednisone equivalent) | Any | 27 (84.4) | 269 (58.7) | **0.004** |
|  | 30-day cumulative steroid dosage (median, IQR)^c^ | 533, 240-1066 | 533, 210-1300 | 0.519 |
|  | Peak dose of  ≤ 1 mg/kg/day^d^ | 8 (29.6) | 78 (29.1) | 0.954 |
|  | Peak dose of  >1 mg/kg/day^d^ | 19 (70.4) | 190 (70.9) |  |
| Previous chest radiotherapy | | 8 (25.0) | 88 (19.2) | 0.425 |
| History of HCT | None | 19 (59.4) | 247 (53.9) | 0.719 |
|  | Autologous | 12 (37.5) | 202 (44.1) |  |
|  | Allogeneic | 1 (3.1) | 9 (2.0) |  |
| History of CAR-T therapy | | 2 (6.3) | 34 (7.4) | 1.000 |
| **RVI clinical course** | | | | |
| Pathogen | RSV | 15 (46.9) | 217 (47.4) | 0.929 |
|  | Influenza | 17 (53.1) | 239 (52.2) |  |
|  | RSV + influenza | 0 (0.0) | 2 (0.4) |  |
| Respiratory viral coinfection (during ± 2 weeks) | | 4 (12.5) | 81 (17.7) | 0.454 |
| Year of infection | 2016 | 5 (5.6) | 40 (8.7) | 0.700 |
|  | 2017 | 4 (12.5) | 94 (20.5) |  |
|  | 2018 | 6 (18.8) | 67 (14.6) |  |
|  | 2019 | 8 (25.0) | 107 (23.4) |  |
|  | 2020 | 3 (9.4) | 50 (10.9) |  |
|  | 2021 | 1 (3.1) | 35 (7.6) |  |
|  | 2022 | 5 (15.6) | 65 (14.2) |  |
| Time period of infection | Pre COVID-19 era (1/2016-2/2020) | 26 (81.3) | 350 (76.4) | 0.532 |
|  | COVID-19 era (3/2020-12/2022) | 6 (18.8) | 108 (23.6) |  |
| Site of infection at presentations | URI | 7 (21.9) | 296 (64.6) | **<0.001** |
|  | LRI | 25 (78.1) | 162 (35.4) |  |
| Progression to LRI (among URI)^e^ | | 2 (28.6) | 17 (5.7) | **0.014** |
| Total LRI (presentation and progression) | | 27 (84.4) | 179 (39.1) | **<0.001** |
| LRI type^f^ | Probable | 14 (51.9) | 156 (87.2) | **<0.001** |
|  | Laboratory confirmed | 13 (48.1) | 23 (12.8) |  |
| RVI symptoms | Cough | 25 (78.1) | 403 (88.0) | 0.162 |
|  | Fever | 9 (28.1) | 241 (52.6) | **0.007** |
|  | Shortness of breath | 13 (40.6) | 149 (32.5) | 0.347 |
|  | Rhinorrhoea | 3 (9.4) | 145 (31.7) | **0.008** |
|  | Nasal congestion | 5 (15.6) | 146 (31.9) | 0.054 |
|  | Fatigue | 16 (50.0) | 133 (29.0) | **0.013** |
|  | Sore throat | 1 (3.1) | 72 (15.7) | 0.053 |
|  | Chills | 3 (9.4) | 78 (17.0) | 0.260 |
|  | Headache | 1 (3.1) | 53 (11.6) | 0.237 |
|  | Nausea/vomiting | 5 (15.6) | 50 (10.9) | 0.387 |
|  | Myalgia | 0 (0.0) | 46 (10.0) | 0.060 |
|  | Diarrhoea | 1 (3.1) | 37 (8.1) | 0.498 |
|  | Chest pain | 3 (9.4) | 30 (6.6) | 0.467 |
|  | Arthralgia | 1 (3.1) | 13 (2.8) | 1.00 |
| Hypoxia at presentation (≤92%) in room air^g^ | | 14 (46.7)^h^ | 35 (8.1)^i^ | **<0.001** |
| Nosocomial infection | | 5 (15.6) | 15 (3.3) | **0.007** |
| Bronchoscopy | | 15 (46.9) | 27 (5.9) | **<0.001** |
| Lymphopenia (<200 cells/ml) | | 18 (56.3) | 42 (9.2) | **<0.001** |
| Neutropenia (<500 cells/ml) | | 7 (21.9) | 21 (4.6) | **0.001** |
| Lymphopenia & neutropenia | | 6 (18.8) | 11 (2.4) | **<0.001** |
| Elevated creatinine (≥1.2 mg/dl) | | 13 (40.6) | 133 (29.0) | 0.166 |
| Antiviral therapy | Any therapy | 29 (90.6) | 361 (78.8) | 0.109 |
|  | Ribavirin | 12 (37.5) | 130 (28.4) | 0.272 |
|  | Oseltamivir | 18 (56.3) | 227 (49.6) | 0.465 |
|  | IVIG | 9 (28.1) | 58 (12.7) | **0.028** |
| Antiviral timing from symptom onset | No treatment | 3 (9.4) | 97 (21.2) | 0.168 |
|  | Within 48 h | 8 (25.0) | 131 (28.6) |  |
|  | After 48 h | 21 (65.6) | 230 (50.2) |  |
| **RVI outcomes** | | | | |
| Hospital admission | Any | 29 (90.6) | 251 (54.8) | **<0.001** |
|  | Secondary to RVI | 18 (56.3) | 214 (46.7) | 0.297 |
|  | Length of stay (median, IQR)^j^ | 14, 6-26 | 5, 3-8 | **<0.001** |
| ICU admission | | 15 (46.9) | 23 (5.0) | **<0.001** |
| Oxygen requirement (maximal) | None | 7 (21.9) | 325 (71.0) | **<0.001** |
|  | Nasal cannula | 6 (18.8) | 92 (20.1) |  |
|  | Face mask | 1 (3.1) | 7 (1.5) |  |
|  | HFNC | 4 (12.5) | 16 (3.5) |  |
|  | BiPAP | 5 (15.6) | 10 (2.2) |  |
|  | Mechanical ventilation | 9 (28.1) | 8 (1.7) |  |
| Follow-up duration, days (median, IQR) | | 28, 12-53 | 90, 90-90 | **<0.001** |

^a^Data are no. (%) unless otherwise specified; ^b^n=489; ^c^n=294; ^d^n=295; ^e^n=303; ^f^n=206; ^g^n=462; ^h^n=30; ^i^n=432; ^j^n=280.

Abbreviations: BiPAP, bilevel positive airway pressure; CAR-T, chimeric antigen receptor T-cell therapy; COVID-19, coronavirus disease 2019; HCT, hematopoietic stem cell transplantation; HFNC, high-flow nasal cannula; HM, hematologic malignancy; ICU, intensive care unit; IQR, interquartile range; IVIG, intravenous immunoglobulin; LRI, lower respiratory tract infection; RSV, respiratory syncytial virus; RVI, respiratory virus infection; SD, standard deviation; URI, upper respiratory tract infection.

| **Table S4. Multivariable logistic regression model of the independent predictors of developing LRI among different cancer patients.** | | | |
| --- | --- | --- | --- |
|  |  |  |  |
| **Independent predictor** | **aOR** | **95% CI** | ***p*-value** |
| **a) Lymphoma patients** |  |  |  |
| Age ≥ 65 | 2.74 | 1.41 - 5.35 | **0.003** |
| Lymphopenia (<200 cells/ml) | 4.95 | 1.73 - 14.20 | **0.003** |
| Previous chest radiotherapy | 2.53 | 1.07 - 5.99 | **0.035** |
| Pathogen* |  |  |  |
| RSV | 1.99 | 1.04 - 3.81 | **0.039** |
| Influenza | Reference |  |  |
| Respiratory viral coinfection | 2.30 | 1.03 - 5.10 | **0.041** |
| **b) Multiple myeloma patients** |  |  |  |
| Lymphopenia (<200 cells/ml) | 3.38 | 1.55 - 7.37 | **0.002** |
| Elevated creatinine (≥1.2 mg/dl) | 1.80 | 1.09 - 2.97 | **0.022** |
| Steroid use at RVI diagnosis (within 30 days) | 2.79 | 1.56 - 4.97 | **0.001** |

| * Two patients who had both RSV and influenza infections were excluded from the analysis.  Abbreviations: aOR = adjusted odds ratio; 95% CI = 95% confidence interval. |
| --- |
